# Studies on the Incompatibility between *Bulbus fritillariae* and *Radix aconiti praeparata* Based on the P-gp

**DOI:** 10.1155/2021/8351717

**Published:** 2021-10-05

**Authors:** Weijun Liu, Ling Wei, Yoshikatsu Kanai, Xin He

**Affiliations:** ^1^Tianjin Key Laboratory of Acute Abdomen Disease Associated Organ Injury and ITCWM Repair, Tianjin Nankai Hospital, Tianjin 300100, China; ^2^School of Chinese Materia Medica, Guangdong Pharmaceutical University, Guangzhou, Guangdong 510006, China; ^3^Department of Bio-System Pharmacology, Graduate School of Medicine, Osaka University, Suita, Osaka 565-0871, Japan

## Abstract

*Bulbus fritillariae* and *Radix aconiti praeparata* are an incompatible herbal pair in the traditional Chinese medicine theory “eighteen incompatible medicaments,” and they should not be used simultaneously in clinical treatment for safety. This study aimed to investigate the incompatibility mechanism between *Bulbus fritillariae* and *Radix aconiti praeparata* based on their interaction with P-glycoprotein (P-gp). The interaction between *Bulbus fritillariae* and *Radix aconiti praeparata* during *in vitro* decocting as well as *in vivo* absorption was investigated by determining the dry extract yield and by rat single-pass intestinal perfusion (SPIP) model. Inhibition of different species of *Bulbus fritillariae* on P-gp function was examined using the SPIP model. The mRNA and protein expression of P-gp was determined by PCR and western blotting. The active ingredients of *Bulbus fritillariae* were predicted and screened for inhibiting P-gp activity by Schrodinger's molecular docking and MDR1-MDCK cell transport study, respectively. Mediation of monoester alkaloids in *Radix aconiti praeparata* by P-gp was predicted and examined using Schrodinger's molecular docking and SPIP experiment, respectively. In the results, when *Radix aconiti praeparata* was combined with *Bulbus fritillariae*, the toxic ingredient benzoylmesaconine in *Radix aconiti praeparata* displayed higher intestinal permeability, whereas the toxic ingredients showed no significant difference during the *in vitro* decoction process. *Bulbus fritillariae thunbergii* inhibited both the P-gp function and expression; in contrast, *Bulbus fritillariae cirrhosae* inhibited the function only. Alkaloids including peimine, peimisine, and imperialine were the active ingredients for inhibiting P-gp activity. Benzoylmesaconine in *Radix aconiti praeparata* was the substrate of P-gp.

## 1. Introduction

The traditional Chinese medicine theory “eighteen incompatible medicaments” refers to the incompatibility of the Chinese herbs, and it indicates that pairs of herbs which are mutually incompatible should not be used simultaneously. *Bulbus fritillariae* and *Radix aconiti praeparata* are one incompatible herbal pair as recorded in “eighteen incompatible medicaments.” *Bulbus fritillariae*, a plant bulb from Liliaceae family, is a well-known and “medicinal and edible” herb. It has been used for centuries to treat cough, phlegm, and inflammation. There are approximately 25 species of *Bulbus fritillariae*, all of which are called “Beimu” in commercial markets, and they can differ in price by more than 100-fold [1]. Among them, *Bulbus fritillariae thunbergii* and *Bulbus fritillariae cirrhosae* have the highest medicinal value, although they contain different ingredients. Alkaloids are the main ingredients of *Bulbus fritillariae thunbergii* and *Bulbus fritillariae cirrhosae*. *Bulbus fritillariae thunbergii* contains peimine, peiminine, and peimisine. *Bulbus fritillariae cirrhosae* contains peimisine and imperialine [2].

*Radix aconiti*, a plant root from Ranunculaceae family, is also a well-known and valuable herb. It has been utilized for centuries, and has the effects of anti-inflammatory, analgesia, and blood activating. The effective and toxic ingredients in *Radix aconiti* are aconitum alkaloids [3, 4]. Aconitum alkaloids could be divided into diester alkaloids, monoester alkaloids, and lipid alkaloids according to their structure. After processing of crude aconite roots, most of the diester-diterpenoid alkaloids with high toxicity convert to the monoester form with low toxicity. The remaining diester-diterpenoid alkaloids will further be hydrolyzed to monoester-diterpenoid alkaloids during the decoction process.

The extent of drug absorption in the gastrointestinal tract highly affects its pharmacological and toxicological effects. Transporters are increasingly recognized as a key determinant in the intestinal absorption of drugs [5]. Among these transporters, P-glycoprotein (P-gp), encoded by ABCB1 (also called multiple drug resistant 1, MDR1) gene, is one of the most well-studied and the best-characterized drug transporters belonging to the ATP-binding cassette (ABC) transporter family. It is present in the apical membrane of intestinal epithelia cells where it regulates the intestinal absorption by mediating the efflux. Inhibition of P-gp may cause marked changes in the absorption profile of the affected drugs [6] and thus results in clinically significant drug-drug interaction (DDI) as well as herb-herb interaction (HHI).

Recent studies have proved that the combination between *Bulbus fritillariae* and *Radix aconiti praeparata* has adverse reactions [7–9]. The toxicity of *Radix aconiti praeparata* was increased. However, the mechanism is unclear. Studies have shown that *Bulbus fritillariae thunbergii* reverses the multidrug resistance (MDR) by inhibiting the expression of P-gp in MDR tumor cells [10, 11]. To determine whether the incompatibility between *Bulbus fritillariae* and *Radix aconiti praeparata* could be based on the HHI mediated by P-gp, we design the present study to investigate the incompatibility in the decoction process or intestinal absorption process. Whether *Bulbus fritillariae thunbergii* or *Bulbus fritillariae cirrhosae* inhibits the P-gp function or expression is explored. The active ingredients of this effect are screened. Whether monoester alkaloids in *Radix aconiti praeparata* are the P-gp substrate is explored. By this research, we will achieve a better understanding of the incompatibility mechanism between *Bulbus fritillariae* and *Radix aconiti praeparata* and reveal its material basis.

## 2. Materials and Methods

### 2.1. Plant Material

**Figure 1:**
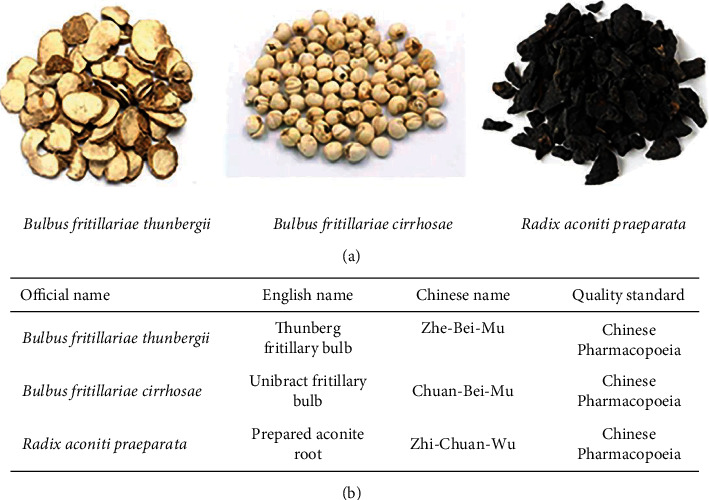
Information of the incompatible herbal pair. (a) The representative photograph of herbs. (b) The list of herbal names.

Herbs of *Bulbus fritillariae thunbergii*, *Bulbus fritillariae cirrhosae*, and *Radix aconiti praeparata* (Figure 1(a)) were obtained from Pharmacy Department in Tianjin Nankai Hospital (China). Botanical identity was authenticated by the executive manager of Pharmacy Department in Tianjin Nankai Hospital. They were stored at room temperature. Voucher specimens with storage codes were deposited at the Pharmacy Department in Tianjin Nankai Hospital. The herbal names were checked in accordance with Chinese Pharmacopoeia (Figure 1(b)).

### 2.2. Chemicals and Drugs

Digoxin (≥98%), cyclosporine A (≥98%), and verapamil (≥98%) were provided from the National Institute for the Control of Pharmaceutical and Biological Products (Beijing, China). Peimine, peiminine, peimisine, imperialine, adenosine, glucose, benzoylaconine, benzoylmesaconine, and benzoylhypacoitine were purchased from Zelang Co., Ltd. (Nanjing, China). HPLC-grade reagents were obtained from Concord Co., Ltd. (Tianjin, China). Ultrapure water was prepared by a Milli-Q Synthesis system (Millipore, Billerica, USA). All other reagents and solvents were of analytical grade or better.

### 2.3. Animals

Male SD rats (weight 210–230 g; age 2 months) were provided by HFK Bioscience Co., Ltd. (Beijing, China). The rats were maintained on a 12 h light-dark cycle and fasted 12 h before the experiment with free access to water. The Animal Ethical and Welfare Committee in Tianjin Nankai Hospital (Approval no. NKYY-DWLL-2020-002) approved the animal study. The animal study adhered to the principles of laboratory animal use and care (NIH publication #85-23, revised in 1985).

### 2.4. Preparation of the Extracts

*Bulbus fritillariae thunbergii* or *Bulbus fritillariae cirrhosae* was extracted in water by reflux method at 90°C for 2 h. This process was repeated three times. The decoction was filtrated by gauze and dried to *Bulbus fritillariae* extract. *Radix aconiti praeparata* was extracted in water by reflux method at 90°C for 2 h. This process was repeated three times. The decoction was filtrated by gauze and dried to *Radix aconiti praeparata* extract. *Radix aconiti praeparata* + *Bulbus fritillariae thunbergii* or *Bulbus fritillariae cirrhosae* in a 1 : 1 (w/w) ratio was extracted in water by reflux method at 90°C for 2 h. This process was repeated three times. The decoction was filtrated by gauze and dried to *Radix aconiti praeparata* + *Bulbus fritillariae* extract. The dry extract yield was determined.

### 2.5. Preparation of the Active Ingredients in *Bulbus fritillariae*

The *Bulbus fritillariae* extract was dissolved in water and the solution flew through D101-type macroporous resin. The resin was cleansed by water, 30% ethanol, and 95% ethanol in sequence. The water eluate was mixed with ethanol and then deposited. The deposit was dried, and the polysaccharide was got. The content was determined by anthrone colorimetry. The 30% ethanol eluate was dried, and nucleoside was got. The content was determined by ultraviolet spectrophotometry; the 95% ethanol eluate flew through 732-type cation exchange resin. The resin was cleansed by water and 0.5 M ammonia ethanol in sequence. The ammonia ethanol eluate was dried, and alkaloid was got. The content was determined by acid dye colorimetry. The contents of alkaloid, nucleoside, and polysaccharide in *Bulbus fritillariae thunbergii* extracts were 53.13%, 32.23%, and 27.69%, respectively. Those in *Bulbus fritillariae cirrhosae* extracts were 32.62%, 27.83%, and 23.73%, respectively. The active ingredients were quantified.

### 2.6. Single-Pass Intestinal Perfusion (SPIP)

Rats were anaesthetized by an intraperitoneal injection of urethane and then placed on a heated pad. The small intestine was exposed by surgery. 10 cm of the ileum segment was ligated and cannulated with glass tubing for perfusion. Caroline et al. [12] reported that the level of P-gp expression in rat ileum was the highest. In order to better reflect the characteristics of P-gp, we selected the ileum segment. The cannulated segment was rinsed with saline (37°C), and attached to the perfusion assembly consisted of a syringe pump and a 50 ml syringe. The selected ileum was placed as “U” shape and covered with the gauze wet by saline. The perfusate was collected every 10 min at a flow rate of 0.2 ml/min for 90 min. The perfused intestine segment was removed and opened along the mesentery to measure the area. Samples were frozen immediately and stored at -20°C until analysis. Digoxin, an exclusive substrate of P-gp, was chosen as the FDA guidelines [13]. Cyclosporine A or verapamil, an exclusive inhibitor of P-gp, was selected as the FDA guidelines [13] or Chen et al.'s report [14].

### 2.7. HPLC Analysis

The concentrations of drugs in SPIP experiment were determined by HPLC. A MS-C_18_ column (5 *μ*m, 4.6 mm × 150 mm, Agilent, USA) with an analytical TC-C_18_ guard column (5 *μ*m, 4.6 mm × 12.5 mm, Agilent, USA) was maintained at 30°C. The analytical mobile phase about digoxin consisted of water and acetonitrile in a 75 : 25 (v/v) ratio. Benzoylaconine, benzoylmesaconine, and benzoylhypacoitine consisted of 0.05% acetic acid and methanol in a gradient from 85 : 15 to 55 : 45 (v/v) ratio. The flow rate was 1 mL/min. The injection volume was 10 *µ*L. Digoxin was detected by absorbance at 220 nm and benzoylaconine, benzoylmesaconine, and benzoylhypacoitine at 235 nm.

### 2.8. PCR Analysis

Rats were anaesthetized by an intraperitoneal injection of urethane. The ileum was removed, and rinsed with saline. Then, it was placed on an ice-cold plate, and cut along the mesentery, and the mucosa was gently scraped for intestinal epithelial tissue. Total RNA was extracted from the separated cells clumps using RNAsimple Total RNA kit (Tiangen, China), according to the manufacturer's instructions. RNA concentration was spectrophotometrically quantified. The integrity of RNA was electrophoretically checked on agarose gel stained with ethidium bromide. TIANScript RT kit (Tiangen, China) was used for reverse transcription of total RNA. To quantify the cycle threshold (*Ct*) value, real-time PCR was performed on ABI7500 Real-Time PCR instrument (ABI, USA) using Power SYBR™ Green PCR Master Mix (ABI, USA). Sense and antisense primers used in this analysis were as follows: P-gp, 5'- AACACCCTGGTTGGTGAGAG-3' and 5'-CACCATCAAAACCAGCAATG-3'; *β*-actin, 5'-CCCATCTATGAGGGTTACGC-3' and 5'-TTTAATGTCACGCACGATTTC-3'. *β*-Actin was used for normalization.

### 2.9. Western Blotting Analysis

Rats were anaesthetized by an intraperitoneal injection of urethane. The ileum was removed, and rinsed with saline. Then, it was placed on an ice-cold plate, and cut along the mesentery, and the mucosa was gently scraped for intestinal epithelial tissue. The separated tissue was homogenized by RIPA lysis buffer, and then centrifuged at 12000 g for 5 min at 4°C. The resulting supernatant was got. The concentration of protein was determined by the BCA Assay Protein kit. For gel electrophoresis, 50 *μ*g of total protein of each sample was added in Protein Loading Dye, and denatured for 5 min at 95°C. Samples were separated by electrophoresis in 8% SDS-PAGE, and transferred to a polyvinylidene difluoride (PVDF) membrane. The membrane was blocked with 5% nonfat milk in TBST at room temperature for 2 h. For the detection of the interest proteins, the blots were incubated with the primary antibodies and the secondary antibody. After washing with TBST, the membrane was detected by the enhanced Chemiluminescent HRP Substrate according to the manufacturer's protocol and exposed to X-ray film for up to 5 min. *β*-Actin was used as an internal reference. The optical density (OD) for the band of interest was measured by the Quantity One software (Bio-Rad, USA).

### 2.10. Molecular Docking

The 3D crystal structure of P-gp (PDB ID: 4F4C, 3G60; organism(s): *Caenorhabditis elegans* and *Mus musculus*) was downloaded from the Protein Data Bank (https://www.rcsb.org/). The 2D structures of active ingredients were downloaded from the ChemSpider (http://www.chemspider.com/). The receptor sites of P-gp, the ligand sites of active ingredients, and the receptor-ligand docking were performed by Schrodinger 2017. Parameters involved were selected as the system default settings.

### 2.11. Cell Transport Study

MDR1-MDCK cells, obtained from the College of Pharmaceutical Sciences in Zhejiang University (China), were cultured in DMEM containing 10% FBS, 100 *µ*g/mL-100 U/mL penicillin-streptomycin, and 1% NEAA at 37°C in an atmosphere of 5% CO_2_ and 95% relative humidity. Cells were passaged at 80%–90% conﬂuence. P-gp inhibitors were screened by MDR1-MDCK cell transport study using the calcein-AM kit. Briefly, the cells were seeded in a 96-well black opaque plate and cultured for 48 h. Then, the cells were washed twice with PBS. 100 *µ*L drug and 50 *µ*L culture medium was added and preincubated at 37°C for 10 min. 50 *µ*L 1 *µ*M calcein-AM was added and incubated at 37°C for 1 h. Cold PBS was used to terminate the reaction. 100 *µ*L 0.1% Triton-100 was added for cell permeability. The relative fluorescence unit (RFU) of calcein was determined at EX = 494 nm and EM = 517 nm.

### 2.12. Data Analysis

#### 2.12.1. Effective Permeability Coefficient (*P*_eff_)

*P*_eff_ was calculated by the following equation:

(1) $$\begin{array}{c} P_{\text{eff}}=-Q_{\text{in}}\ln\frac{\left(C_{\text{out}}Q_{\text{out}}/C_{\text{in}}Q_{\text{in}}\right)}{\left(2\pi rL\right)}. \end{array}$$

Here, *Q*_in_ and *Q*_out_ were the flow rate at inlet and outlet, respectively. *C*_in_ and *C*_out_ were the concentration at inlet and outlet, respectively. 2*πrL* was the mass transfer surface area within the intestinal segment, which was assumed to be the area of the cylinder with the radius (*r*) and length (*L*).

#### 2.12.2. Relative Gene Expression (RGE)

RGE was calculated by

(2) $$\begin{array}{c} \text{RGE}=2^{-\left(\Delta CtTS-\Delta CtCS\right)}. \end{array}$$

Here, △*Ct TS* was the difference between the *Ct* of the target gene and reference gene of the test sample and △*Ct CS* was the difference between the *Ct* of the target gene and reference gene of the control sample.

#### 2.12.3. Relative Protein Expression (RPE)

RPE was calculated by

(3) $$\begin{array}{c} \text{RPE}=\frac{\text{OD TB}}{\text{OD RB}}. \end{array}$$

Here, OD TB was the OD of the target band and OD RB was the OD of the reference band.

#### 2.12.4. Relative Fluorescence Rate (RFR)

RFR was calculated by

(4) $$\begin{array}{c} \text{RFR}=\frac{\text{RFU TS}}{\text{RFU CS}}\times 100\%. \end{array}$$

Here, RFU TS was the RFU of the test sample and RFU CS was the RFU of the control sample.

### 2.13. Statistical Analysis

All results were expressed as mean ± SD. The difference between the means was analyzed for significance by *t*-test in SPSS 11.5. The *p*-value was considered statistically significant or highly significant, when less than 0.05 or 0.01.

## 3. Results

### 3.1. HHI between *Bulbus fritillariae* and *Radix aconiti praeparata* in Decoction Process

The toxic ingredients in *Radix aconiti praeparata* are mainly monoester alkaloids, such as benzoylmesaconine, benzoylaconine, or benzoylhypacoitine. No significant differences were observed between the yield of benzoylmesaconine in *Radix aconiti praeparata* extract (15.80 ± 0.40 mg) and *Radix aconiti praeparata* + *Bulbus fritillariae thunbergii* extract (16.47 ± 1.84 mg), between the yield of benzoylaconine in *Radix aconiti praeparata* extract (1.85 ± 0.28 mg) and *Radix aconiti praeparata* + *Bulbus fritillariae thunbergii* extract (1.31 ± 0.60 mg), or between the yield of benzoylhypacoitine in *Radix aconiti praeparata* extract (0.79 ± 0.10 mg) and *Radix aconiti praeparata* + *Bulbus fritillariae thunbergii* extract (0.96 ± 0.51 mg) (*n* = 3). There were also no significant differences between the yield of benzoylmesaconine in *Radix aconiti praeparata* extract (15.80 ± 0.40 mg) and *Radix aconiti praeparata* + *Bulbus fritillariae cirrhosae* extract (17.05 ± 1.33 mg), between the yield of benzoylaconine in *Radix aconiti praeparata* extract (1.85 ± 0.28 mg) and *Radix aconiti praeparata* + *Bulbus fritillariae cirrhosae* extract (2.41 ± 0.61 mg), or between the yield of benzoylhypacoitine in *Radix aconiti praeparata* extract (0.79 ± 0.10 mg) and *Radix aconiti praeparata* + *Bulbus fritillariae cirrhosae* extract (0.94 ± 0.20 mg) (*n* = 3). It illuminated that the *Bulbus fritillariae* could not affect the yield of toxic ingredients-monoester alkaloids in *Radix aconiti praeparata* in the decoction process.

### 3.2. Feasibility of the SPIP Model

**Figure 2:**
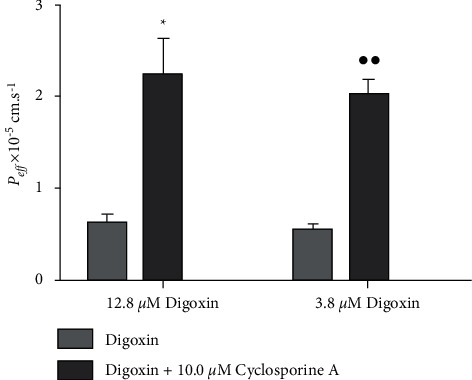
Feasibility of the SPIP model (*n* = 3). All data were presented as the mean ± SD. ^*∗*^*p* < 0.05; ^*∗∗*^*p* < 0.01.

Figure 2 shows an analysis of SPIP model's feasibility. *P*_eff_ of the group given 12.8 *μ*M digoxin with cyclosporine A showed significant differences from the group given only 12.8 *μ*M digoxin (*p* < 0.05). Additionally, *P*_eff_ of the group given 3.8 *μ*M digoxin with cyclosporine A showed highly significant differences from the group given only 3.8 *μ*M digoxin (*p* < 0.01). These results indicated that the P-gp's efflux function was reasonably shown by the SPIP model. A digoxin concentration of 3.8 *μ*M was chosen as the base because it better reflected the observed efflux transport.

### 3.3. HHI between *Bulbus fritillariae* and *Radix aconiti praeparata* in Absorption Process

**Figure 3:**
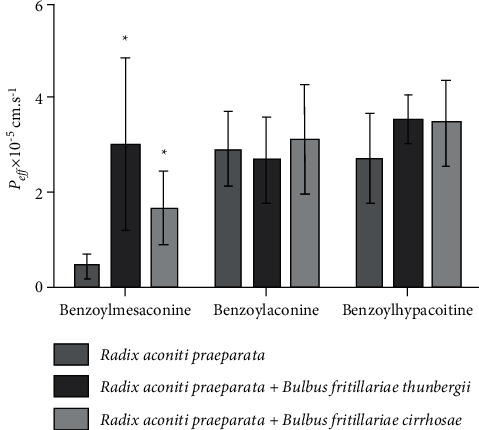
Influence of *Bulbus fritillariae thunbergii* or *Bulbus fritillariae cirrhosae* on the *P*_eff_ of monoester alkaloids in *Radix aconiti praeparata* (*n* = 6). All data were presented as the mean ± SD. ^*∗*^*p* < 0.05.

The influence of *Bulbus fritillariae thunbergii or Bulbus fritillariae cirrhosae* on the *P*_eff_ of monoester alkaloids in *Radix aconiti praeparata* is shown in Figure 3. There were significant differences between the *P*_eff_ of benzoylmesaconine in *Radix aconiti praeparata* extract and *Radix aconiti praeparata* + *Bulbus fritillariae thunbergii* extract (*p* < 0.05). No significant differences were observed between the *P*_eff_ of benzoylaconine or benzoylhypacoitine in *Radix aconiti praeparata* extract and *Radix aconiti praeparata* + *Bulbus fritillariae thunbergii* extract. There were significant differences between the *P*_eff_ of benzoylmesaconine in *Radix aconiti praeparata* extract and *Radix aconiti praeparata* + *Bulbus fritillariae cirrhosae* extract (*p* < 0.05). No significant differences were observed between the *P*_eff_ of benzoylaconine or benzoylhypacoitine in *Radix aconiti praeparata* extract and *Radix aconiti praeparata* + *Bulbus fritillariae cirrhosae* extract. It demonstrated that the absorption extent of benzoylmesaconine, a toxic ingredient, in *Radix aconiti praeparata* was enhanced by *Bulbus fritillariae thunbergii* or *Bulbus fritillariae cirrhosae*.

### 3.4. Inhibition of *Bulbus fritillariae* on P-gp Function

**Figure 4:**
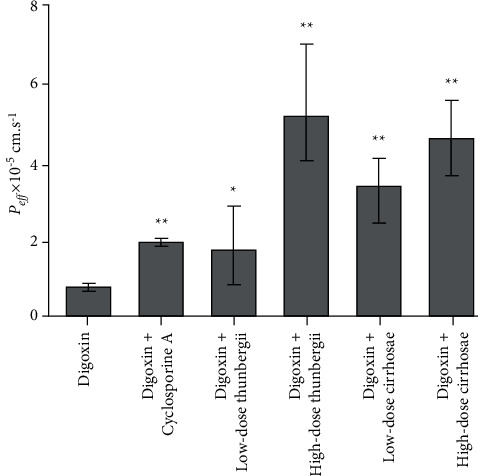
Influence of the *Bulbus fritillariae thunbergii* and *Bulbus fritillariae cirrhosae* on the *P*_eff_ of digoxin (*n* = 6). All data were presented as the mean ± SD. ^*∗∗*^*p* < 0.01; ^*∗*^*p* < 0.05.

The influence of the *Bulbus fritillariae thunbergii* and *Bulbus fritillariae cirrhosae* extracts on the *P*_eff_ of digoxin is shown in Figure 4. The *P*_eff_ of digoxin with cyclosporine A showed highly significant differences from that of digoxin (*p* < 0.01). It proved that the cyclosporine A significantly inhibited the P-gp function in the ileum. There were significant differences between the *P*_eff_ of digoxin and that of digoxin supplemented with low-dose (*p* < 0.05) or high-dose (*p* < 0.01) *Bulbus fritillariae thunbergii* extract. Highly significant differences were observed between the *P*_eff_ of digoxin and that of digoxin supplemented with low-dose or high-dose *Bulbus fritillariae cirrhosae* extract (*p* < 0.01). It demonstrated that the *Bulbus fritillariae thunbergii* and *Bulbus fritillariae cirrhosae* extracts significantly inhibited the P-gp function in the ileum in a concentration dependent manner.

### 3.5. Inhibition of *Bulbus fritillariae* on P-gp Expression

**Figure 5:**
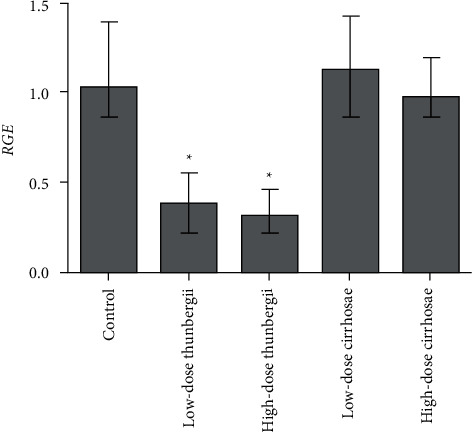
Influence of the *Bulbus fritillariae thunbergii* and *Bulbus fritillariae cirrhosae* on the RGE of mdr1 in the ileum (*n* = 3). All data were presented as the mean ± SD. ^*∗*^*p* < 0.05.

**Figure 6:**
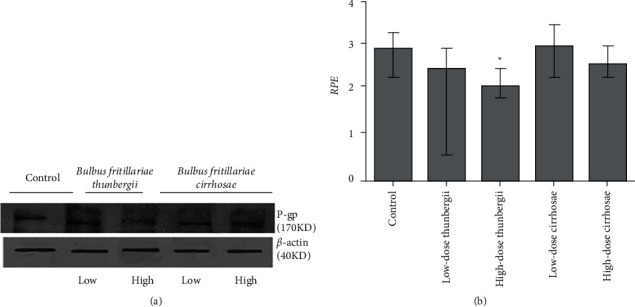
Influence of the *Bulbus fritillariae thunbergii* and *Bulbus fritillariae cirrhosae* on the RPE of P-gp in the ileum (*n* = 3). (a) Representative Western blotting. (b) Semiquantitative densitometric analysis. All data were presented as mean ± SD. ^*∗*^*p* < 0.05.

The influence of the *Bulbus fritillariae thunbergii* and *Bulbus fritillariae cirrhosae* extracts on the RGE of mdr1 and RPE of P-gp in the ileum is shown in Figures 5 and 6(a) and 6(b), respectively. There were significant differences between the RGE of the control group and low-dose or high-dose *Bulbus fritillariae thunbergii* group (*p* < 0.05). On the contrary, RGE showed no significant change in *Bulbus fritillariae cirrhosae* group. It indicated that the *Bulbus fritillariae thunbergii* extract significantly inhibited the mdr1 mRNA expression in the ileum in a concentration-dependent manner, but *Bulbus fritillariae cirrhosae* did not. There were significant differences between the RPE of the control group and high-dose *Bulbus fritillariae thunbergii* group (*p* < 0.05), except for no significant differences in low-dose group. On the contrary, RPE showed no significant change in *Bulbus fritillariae cirrhosae* group. It hinted that the *Bulbus fritillariae thunbergii* extract significantly inhibited the P-gp expression in the ileum in a concentration-dependent manner, but *Bulbus fritillariae cirrhosae* did not.

### 3.6. Feasibility of Cell Transport Study by the Calcein-AM Kit

**Figure 7:**
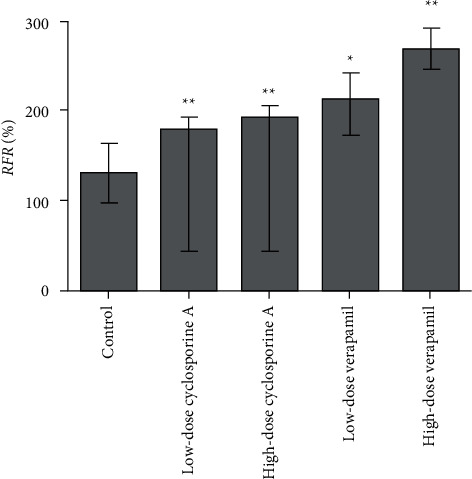
Feasibility of the calcein-AM kit (*n* = 3). All data were presented as the mean ± SD. ^*∗∗*^*p* < 0.01; ^*∗*^*p* < 0.05.

Figure 7 shows an analysis of calcein-AM kit's feasibility. The RFR of the low-dose or high-dose cyclosporine A group showed highly significant differences from the control group (*p* < 0.01). Additionally, the RFR in the low-dose (*p* < 0.05) or high-dose (*p* < 0.01) verapamil group showed significant differences from the control group. These results indicated that these two inhibitors inhibited P-gp's function by calcein-AM kit in a concentration-dependent manner. The method of this kit was reliable, and the data of this test was effective.

### 3.7. Active Ingredients for Inhibiting P-gp Activity

**Figure 8:**
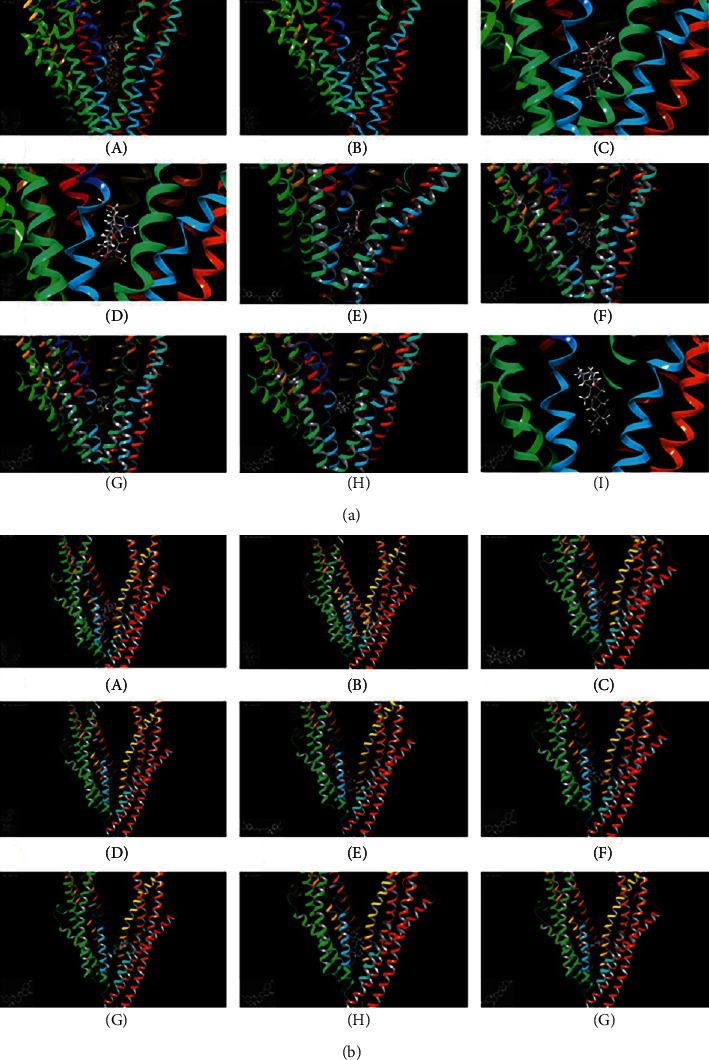
Schrodinger's molecular docking diagram of the active ingredients binding with P-gp. (a) 4F4C and (b) 3G60. (A) Digoxin; (B) benzoylmesaconine; (C) benzoylaconine; (D) benzoylhypacoitine; (E) verapamil; (F) peimine; (G) peiminine; (H) imperialine; (I) peimisine.

**Figure 9:**
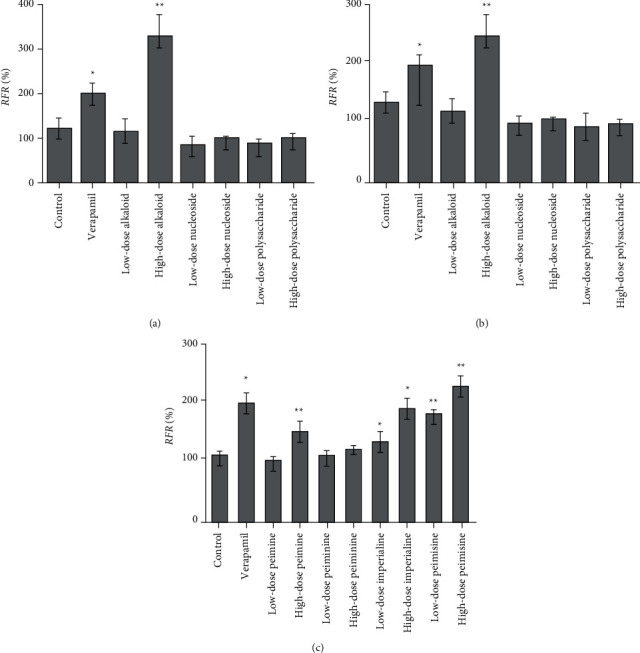
Influence of the active ingredients on the RFR of calcein (*n* = 3). (a) The active portions of *Bulbus fritillariae thunbergii*; (b) the active portions of *Bulbus fritillariae cirrhosae*; (c) the active ingredients of *Bulbus fritillariae*. All data were presented as the mean ± SD. ^*∗*^*p* < 0.05; ^*∗∗*^*p* < 0.01.

In silico prediction of P-gp binding with the active ingredients in *Bulbus fritillariae* was assessed by Schrodinger's molecular docking. In Table 1 and Figure 8, the docking scores of peimine, peimisine, and imperialine with P-gp were comparable with that of verapamil as a positive inhibitor of P-gp. Especially, for the crystal structure of P-gp (4F4C), peimine, peimisine, and imperialine had greater binding potential with P-gp than digoxin, suggesting that they might competitively inhibit P-gp sites. The prediction showed that the peimine, peimisine, and imperialine were potential inhibitors of P-gp. Nonetheless, these active ingredients in *Bulbus fritillariae* needed to be further proved and screened by MDR1-MDCK cell transport study. The results of cell transport study are shown in Figure 9. The verapamil group caused a significant increase in the RFR, compared with the control group (*p* < 0.05) (Figure 9). It proved that the verapamil significantly inhibited the P-gp function in MDR1-MDCK cells. In Figures 9(a) and 9(b), for the alkaloid, there were highly significant differences between the *RFR* of the control group and high-dose *Bulbus fritillariae thunbergii* group (*p* < 0.01), except for no significant differences in low-dose group. Highly significant differences were observed between the RFR of the control group and high-dose *Bulbus fritillariae cirrhosae* group (*p* < 0.01), except for no significant differences in low-dose group. Conversely, for the nucleoside and polysaccharide, the RFR of the low-dose or high-dose *Bulbus fritillariae thunbergii* or *Bulbus fritillariae cirrhosae* group showed no significant differences from the control group. It demonstrated that the alkaloid of *Bulbus fritillariae thunbergii* or *Bulbus fritillariae cirrhosae* significantly inhibited the P-gp function in MDR1-MDCK cells in a concentration-dependent manner, but the nucleoside and polysaccharide did not. In Figure 9(c), there were highly significant differences between the RFR of the control group and high-dose peimine group (*p* < 0.01), except for no significant differences in low-dose group. Significant differences were observed between the RFR of the control group and low-dose or high-dose imperialine group (*p* < 0.05). There were highly significant differences between the RFR of control group and low-dose or high-dose peimisine group (*p* < 0.01). Nevertheless, the RFR of the low-dose or high-dose peiminine group showed no significant differences from the control group. It showed that the peimine, imperialine, and peimisine significantly inhibited the P-gp function in MDR1-MDCK cells in a concentration-dependent manner, but the peiminine did not.

### 3.8. Monoester Alkaloids in *Radix aconiti praeparata* Mediated by P-gp

**Figure 10:**
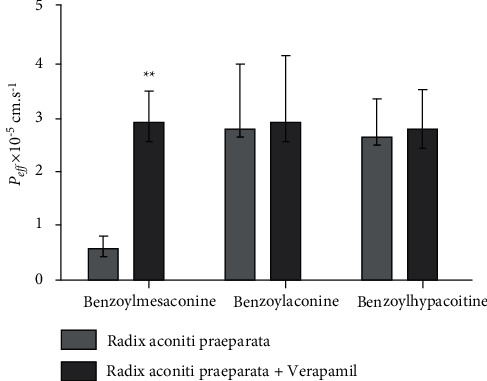
Influence of verapamil on the *P*_eff_ of monoester alkaloids in *Radix aconiti praeparata* (*n* = 6). All data were presented as the mean ± SD. ^*∗∗*^*p* < 0.01.

In silico prediction of P-gp binding with the active ingredients in *Radix aconiti praeparata* was assessed by Schrodinger's molecular docking. In Table 1 and Figure 8, the docking scores of benzoylmesaconine and benzoylaconine with P-gp were comparable with that of digoxin as a positive substrate of P-gp. It showed that the benzoylmesaconine and benzoylaconine were potential substrates of P-gp. However, they needed to be further proved and screened by SPIP experiment. The results of SPIP experiment are shown in Figure 10. In the presence of verapamil, the *P*_eff_ of benzoylmesaconine in *Radix aconiti praeparata* extract had a highly significant change (*p* < 0.01). No significant differences were observed between the *P*_eff_ of benzoylaconine or benzoylhypacoitine in *Radix aconiti praeparata* extract and that supplemented with verapamil. It hinted that benzoylmesaconine was effluxed by P-gp mediation, but benzoylaconine or benzoylhypacoitine was not.

## 4. Discussion

### 4.1. Mechanism of Incompatibility between *Bulbus fritillariae* and *Radix aconiti praeparata*

The “eighteen incompatible medicaments” as an important part of traditional Chinese medicine theory is that a combination of herbs would result in toxic or severe adverse effects, first documented in “Confucians' Duties to Their Parents.” The exploration of its mechanism has been a hot topic in the traditional Chinese medicine research. In recent years, studies have been focusing on the physicochemical changes of the toxic ingredients of herbs [15–18]. During *in vitro* decoction of herbs, the content of original toxic ingredients increases or new toxic ingredients are generated, which could be due to solubilization, hydrotropy, salifying, cosolvency, volatilization, hydrolysis, or oxidation. Besides, the herbs in oral prescription undergo a process of intestinal absorption. Some herbs could affect the process of absorption in the intestinal tract via mucin [19], enzyme, or transporter et al., in which the toxicity could also be enhanced. This study revealed the incompatibility mechanism between *Bulbus fritillariae* and *Radix aconiti praeparata* based on the interaction with P-gp. The results showed that *Bulbus fritillariae* did not affect the yield of toxic ingredients-monoester alkaloids in *Radix aconiti praeparata* when decocting together. The absorption extent of benzoylmesaconine, a toxic ingredient in *Radix aconiti praeparata*, was enhanced by *Bulbus fritillariae thunbergii* or *Bulbus fritillariae cirrhosae*. It meant that the toxicological effect of *Radix aconiti praeparata* would be increased during the intestinal absorption process, but not in the decoction process. *Bulbus fritillariae* was the inhibitor of P-gp. *Bulbus fritillariae thunbergii* inhibited both the P-gp function and expression in the intestine, while *Bulbus fritillariae cirrhosae* inhibited the function of P-gp only. The active ingredients in *Bulbus fritillariae* for inhibiting P-gp activity were alkaloids. *Bulbus fritillariae thunbergii* contained peimine and peimisine; *Bulbus fritillariae cirrhosae* contained peimisine and imperialine. Benzoylmesaconine in *Radix aconiti praeparata* was effluxed by P-gp, which indicated benzoylmesaconine was the substrate of P-gp. This study throws an innovative insight into the theory of “eighteen incompatible medicaments” from the aspect of modern science.

### 4.2. Difference between the Inhibition of P-gp Function and the Inhibition of P-gp Expression by *Bulbus fritillariae*

The ATP-dependent drug efflux transporter P-gp is a 170-kDa membrane protein and it contains two symmetrical halves. Each one has a transmembrane domain (TMD) containing six transmembrane helices and a nucleotide-binding domain (NBD). There are substrate binding sites in the TMD and ATP-binding sites in the NBD. These are the active sites for P-gp function. The inhibition of P-gp function by drugs is the inhibition of active sites. This effect is rapid, direct, and temporary (the effect disappears after withdrawal). The mechanisms behind it include competitive inhibition of substrate binding site, competitive inhibition of ATP-binding site, and noncompetitive inhibition of active regulation site [20, 21]. At the same time, the inhibition of P-gp expression also exists. It is the inhibition of protein level. This effect is slow, indirect, and enduring (the effect lasts for a while after withdrawal). The mechanisms include the regulation on transcription level, translation level, and modification level [22]. We can see that these two types of inhibition are completely different in the phenomenon or mechanism. In the present research, an event was observed: *Bulbus fritillariae thunbergii* inhibited both the P-gp function and expression, but *Bulbus fritillariae cirrhosae* inhibited the function only. Nevertheless, the inhibition mechanism behind it needs to be further analyzed. This will be the subject of our future study.

### 4.3. Two-Sided Combination between *Bulbus fritillariae* and *Radix aconiti praeparata*

The combination between *Bulbus fritillariae* and *Radix aconiti praeparata* could increase the toxicity of *Radix aconiti praeparata* and is not recommended in clinic for safety. On the contrary, there are also some specific examples of the relevant combination in the clinical treatment of diseases, such as arthralgia, senile chronic bronchitis, cough, and pulmonary heart disease [23–25]. In addition, some medical books also describe the prescriptions of the combination between *Bulbus fritillariae* and *Radix aconiti praeparata* (e.g., Huatantang decoction, Huapi paste, and Jinlu pill). This contradiction may be caused by the fact that both active and toxic ingredients in *Radix aconiti praeparata* are monoester alkaloids. The current research told us that the absorption of monoester alkaloid was enhanced when *Bulbus fritillariae* and *Radix aconiti praeparata* were combined. The absorbed monoester alkaloid could exhibit efficacy within a safe dose range while could induce toxicity out of the safe dose range. Overall, it depends on the proportion between *Bulbus fritillariae* and *Radix aconiti praeparata,* and individual differences in patients [26]. The combination between *Bulbus fritillariae* and *Radix aconiti praeparata* shows two sidedness, namely, the enhancement of efficacy as well as the enhancement of toxicity. This explains why the combination of *Bulbus fritillariae* and *Radix aconiti praeparata* belongs to “eighteen incompatible medicaments,” but also appears in some famous prescriptions

## 5. Conclusions

When *Radix aconiti praeparata* was combined with *Bulbus fritillariae*, the toxic ingredient benzoylmesaconine displayed higher intestinal permeability, whereas the toxic ingredients showed no significant difference during the *in vitro* decoction process. *Bulbus fritillariae* was the P-gp inhibitor. *Bulbus fritillariae thunbergii* inhibited both the P-gp function and expression in the intestine, while *Bulbus fritillariae cirrhosae* inhibited the function only. This effect was attributed to alkaloids. The active ingredients of alkaloids in *Bulbus fritillariae thunbergii* were peimine and peimisine, and in *Bulbus fritillariae cirrhosae* were peimisine and imperialine. Benzoylmesaconine in *Radix aconiti praeparata* was the P-gp substrate. This study provides an explanation of the incompatibility mechanism from the perspective of modern science.

## Figures and Tables

**Table 1:** Docking scores of active ingredients binding with P-gp.

Protein	Compound	Docking score (4F4C)	Docking score (3G60)
P-gp	Digoxin	−4.382	−5.833
	Benzoylmesaconine	−5.166	−5.131
	Benzoylaconine	−4.083	−3.654
	Benzoylhypacoitine	−2.534	−2.346
	Verapamil	−6.081	−7.707
	Peimine	−6.617	−5.302
	Peiminine	−3.877	−2.744
	Imperialine	−6.389	−5.379
	Peimisine	−4.597	−4.442

## Data Availability

The data used to support the findings of this study are available from the corresponding author upon request.

## Abbreviations

P-gp: P-glycoprotein
MDR: Multiple drug-resistant
ABC: ATP-binding cassette
DDI: Drug-drug interaction
HHI: Herb-herb interaction
SPIP: Single-pass intestinal perfusion
*Ct*: Cycle threshold
PVDF: Polyvinylidene difluoride
OD: Optical density
RFU: Relative fluorescence unit
*P*_eff_: Effective permeability coefficient
RGE: Relative gene expression
RPE: Relative protein expression
RFR: Relative fluorescence rate
TMD: Transmembrane domain
NBD: Nucleotide-binding domain.
